# The rise of hip research in the *Journal of Experimental Orthopedics*

**DOI:** 10.1002/jeo2.70233

**Published:** 2025-04-13

**Authors:** Sufian S. Ahmad

**Affiliations:** ^1^ Department of Orthopaedic Surgery Hannover Medical School Hannover Germany

**Abbreviations**
JEO: Journal of Experimental Orthopäedics
2D: two‐dimensional
THA: total hip arthroplasty

The past few years have brought significant success to the *Journal of Experimental Orthopaedics* (JEO). The impact factor has continuously increased, and the journal has successfully attracted outstanding submissions of original clinical and basic science research across all areas of orthopaedics. Today, JEO's position among the leading orthopaedic journals is indisputable, and the impact of the published research is clearly evident.

We are pleased to highlight JEO's commitment to the hip joint. Numerous outstanding papers have been accepted, and several studies have undoubtedly transformed our understanding of this area.

Here is a brief overview of some of the key conclusions from the most recently published research:

The significance of the medial circumflex artery as the primary blood supply to the femoral head is well established. A recent study has also highlighted its role in the blood supply to extra‐articular structures of the hip, including the greater trochanter [4].

It has been shown that the presence of a CAM deformity at the head‐neck junction of the femur is likely to influence the measurement of femoral antetorsion, and that three‐dimensional (3D) measurement techniques are more accurate than two‐dimensional (2D) methods [8].

An interesting experimental study demonstrated the ability to acquire information about muscle tension and direction during total hip arthroplasty (THA) through the use of an instrumented trial head during the procedure [2].

It was shown that normal values of pelvic obliquity in a standing position was between 0° and 5.6° (median 2.0°) in the normal asymptomatic population [6].

A further interesting study showed no difference in early complication rates after implantation of short versus conventional stems [1].

In an evaluation of 3D femoral head translation in dysplastic hips, it was found that instability was present in both borderline and truly dysplastic hips. Additionally, the results indicated a trend toward a greater degree of instability in borderline dysplastic hips [7].

Another study investigating hip dysplasia demonstrated a paradox in pelvic tilt, indicating that pelvic tilt contributes to the phenotype of hip dysplasia and ‘adds to’ or exacerbates the pathology, particularly in cases of posterolateral dysplasia [3].

It was also found that hormone replacement therapy is not associated with an increased risk of thromboembolism after THA [5].

These conclusions, drawn from just a small subset of articles, highlight the extensive range of hip research, which includes everything from hip preservation surgery to revision arthroplasty.

We encourage potential authors to submit their best research to JEO and want to emphasise that JEO is just as much a hip journal as it is one focused on shoulder, knee, foot and ankle and basic science topics.

We also invite readers to stay tuned for a series of upcoming exceptional articles that will address the most pressing and current topics in hip surgery.

## CONFLICT OF INTEREST STATEMENT

The author declares no conflict of interest.

## FUNDING INFORMATION

None

## ETHICS STATEMENT

This article did not involve any methodology; therefore, no ethical approval was necessary.
